# The influence of stenosis severity and geometric orientation on atherosclerotic progression: A fluid-structure interaction study in the carotid artery

**DOI:** 10.1007/s13246-026-01784-6

**Published:** 2026-09-14

**Authors:** Harshawardhan Patil, Alexandrina Untaroiu

**Affiliations:** Department of Mechanical Engineering, Virginia Tech, Blacksburg, VA 24061 USA

**Keywords:** Cardiovascular flows, Wall shear stress, Disturbed flow, Atherosclerotic progression, Mooney–Rivlin hyperelasticity

## Abstract

Atherosclerosis remains the leading global cause of mortality, and its progression is strongly influenced by the local hemodynamic environment. Low Wall Shear Stress (WSS) is widely recognized as a promoter of atherosclerosis, yet the combined influence of stenosis severity and its geometric orientation on disease progression in anatomically realistic geometries has not been systematically examined. This study assesses the risk of atherosclerotic progression in a carotid artery using one-way Fluid–Structure Interaction (FSI). Four stenosis levels were simulated (0, 30, 60, and 80%), along with three angular orientations of the stenotic apex relative to the ICA wall. Blood flow was modeled using the *k-ω * SST turbulence model, and arterial walls were represented using a Mooney–Rivlin hyperelastic formulation. For 0% and 30% stenosed cases, regions of low Time-Averaged WSS (TAWSS <= 0.4 Pa) appeared in the ICA. In contrast, the 60% and 80% stenoses produced markedly elevated TAWSS at the stenosis throat, ranging from 20–60 Pa. Arterial wall deformation of the 0% and 30% cases was observed around 0.8 mm, while the 60% and 80% stenoses exhibited prominent foci of high deformation upto 2.1 mm. The presence of disturbed flow dynamics and their resulting atheroprogression risk was assessed through the interplay of Reynolds number, nondimensional WSS, nondimensional Turbulent Kinetic Energy (TKE) and the Oscillatory Shear Index (OSI). At 30% severity, the upstream-tilted stenosis intensified early-stage progression indicators, whereas at 60% severity, the downstream-tilted orientation increased plaque vulnerability. The study demonstrates a comparative framework to rank relative risk of atheroprogression using the disturbed flow signatures and wall mechanics observed across the 8 geometries.

## Introduction

Atherosclerosis is an irreversible, chronic disease affecting millions of people worldwide and the leading cause of death globally. In 2019, 55.5 million new cases were registered globally, with an estimated 17.9 million deaths worldwide [1, 2]. It is characterized as a geometrically focal, complex disease dependent on the histology of arterial tissue, hemodynamic forces at the arterial wall, and multi-modal biomarkers [1, 3, 4]. There is a broad consensus that Wall Shear Stress (WSS) is one of the most significant biomechanical parameters for the initiation and progression of atherosclerotic stenoses [5, 6, 7, 8].

With the advent of numerical methods for a wide range of physical phenomena, evaluation of arterial biomechanics numerically has emerged as a viable alternative [1, 3, 9]. Varghese et al. [10] investigated pulsatile flow for idealized stenotic geometries with four Reynolds-Averaged Navier Stokes (RANS) turbulence models, and found that the low Reynolds * k - ω * turbulence model showed better agreement with experimental results by [11, 12]. For larger arteries with a high shear rate, it is also demonstrated that a Newtonian rheology model for blood is acceptable [13, 14, 15, 16].

Geometrical differences in arterial curvature and undulations directly influence flow dynamics, and may differentially favor disease progression [17]. Given the frequent occurrence of stenoses in the carotid artery, this study focuses on Carotid Artery Stenoses (CAS) [18]. Kaazempur-Mofrad et al. [19] showed a correlation between intimal thickening and plaque location with low and oscillatory wall shear stress. Katranas et al. [8] analyzed whether WSS correlates with plaque morphology, curvature and stiffness, and found that plaque segments had lower WSS than adjacent normal segments.

Younis et al. [20] investigated geometric differences such as bulb size, bifurcation angle, curvature for insights on atherogenesis. They found that geometry is the dominant determinant of the local hemodynamic environment compared to inflow waveform or viscosity model. Notably, they concluded that regions of high cyclic strain and low, oscillatory shear may colocalize.

Fluid Structure Interactions (FSI) have also gained prominence in demonstrating the influence of wall mechanics on the hemodynamic stresses and plaque progression, such as in [21, 22, 23, 24, 25]. Shakya et al. [25] analyzed the effect of stenosis severity in the context of 2-way FSI by comparing 6 levels of stenosis at 4 anatomically distinct sites within the carotid artery. They concluded that while studying hemodynamic response of arteries with multiple stenoses, the effect of one stenosis on another should also be considered [25].

Yang et al. [26] conducted an in-vivo validation study for 2-way FSI, for 14 patients with a total of 32 scan pairs. While they concurred that plaque initiation and early-stage progression evolves in low WSS regions, they argued that the more advanced stages of plaque progression correlate positively with WSS and negatively with plaque wall stress. Their study challenges the accepted mechanism of low WSS driven atheroprogression for advanced stenosis stages.

Holzgapfel et al. [24] reviewed the state-of-the-art for patient-specific CFD and FSI simulations. They note that the most quoted rupture threshold is 300 kPa, however, there is a need to develop a more location and tissue-specific threshold, and a proper failure criterion. Lopes et al. [23] noted that it may be insufficient to assume isotropic linear elasticity of arterial walls due to their non-homogeneous and visco-elastic nature, noting that the preferred choice for capturing mechanical behavior of both healthy and stenosed vessels was hyperelastic models [23].

The works of [21, 22] observe that rigid-wall models overestimate wall shear stresses. Lopes et al. [22] observed that while the effect of varying the arterial stiffness may be minimal, omitting compliance entirely leads to overestimation of velocity magnitudes and shear stresses. Moradicheghamahi et al. (2019) [21] report upto 20% higher maximum shear stress for their rigid wall case. In light of this, it is acknowledged that our study, which employs a 1-way FSI approach, sits at the upper bound of physiologic athero-promotive hemodynamics and wall mechanics.

Given the review of the literature above, it is also pertinent to note that, the influence of stenosis severity in simultaneity with change in orientation of the stenosis in anatomical geometries remains unexplored to the best of our knowledge. In this study, we bridge that gap by exploring the controlled effect of stenosis severity and its geometric orientation on athero-promoting hemodynamics and related wall mechanics. We perform a 1-way FSI analysis on a patient-specific carotid artery with a comparison between 0(healthy), 30, 60 and 80% stenosed cases. 3 levels of angular orientations are considered for the 30% and 60% cases [27]. In addition to elucidating established metrics such as TAWSS and OSI, we also elucidate the interplay between Reynolds Number (Re), nondimensional WSS and non-dimensional Turbulent Kinetic Energy to shed light on the occurrence of persistent flow disturbances.

## Materials and methods

**Fig. 1:**
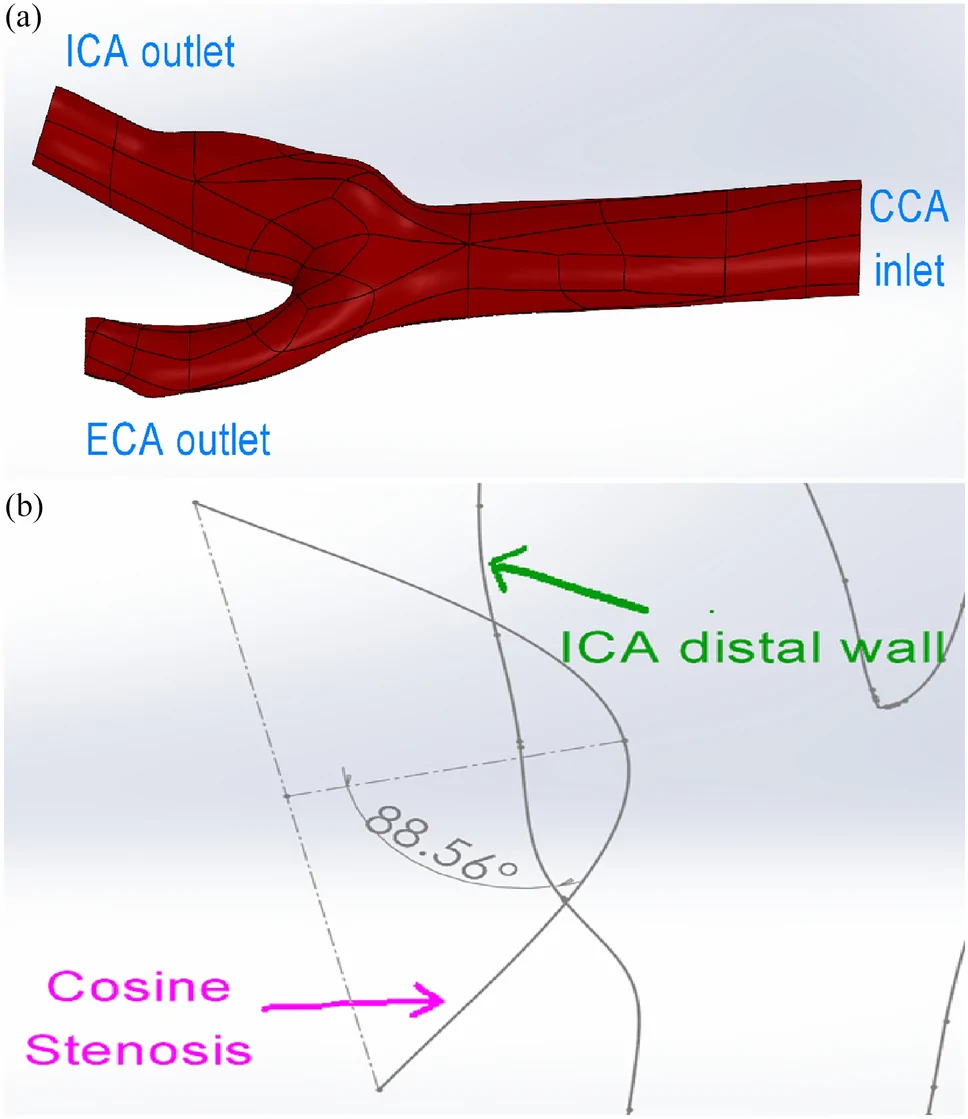
Schematic showing **a** geometric inlet and outlets **b** stenosis encroachment

The CAD Geometry was adapted from the GrabCAD community [28] and has been used in previously published works [21, 22, 25]. The fluid geometry has 1 inlet, belonging to the Common Carotid Artery (CCA), and 2 outlets, which belong to the Internal Carotid Artery (ICA) and External Carotid Artery (ECA) respectively. The ICA and ECA diverge from a small region known as the carotid sinus. The hydraulic diameters of the inlet and outlets are summarized in Table 1, whilst an unstenosed geometry with the inlets and outlets is depicted in Fig. 1a [27].

**Table 1:** Inlet and outlets diameters for ends of the geometry used

End	Hydraulic diameter [mm]	Area [mm^2^]
CCA inlet	6.90	37.48
ICA outlet	4.77	17.94
ECA outlet	3.33	8.74

The model needed to be shelled into a thin-walled vessel for the FEA simulation. To enable successful shelling in SolidWorks 2024, the original CAD model was scaled by a factor of 1.1. This scaled geometry falls within physiological bounds reported in literature [29], and the dimensions provided in Table 1 and the depiction in Fig. 1a is consistent with the same. The wall thickness of the shell is set to 0.7 mm [30].

### Stenosis creation

**Fig. 2:**
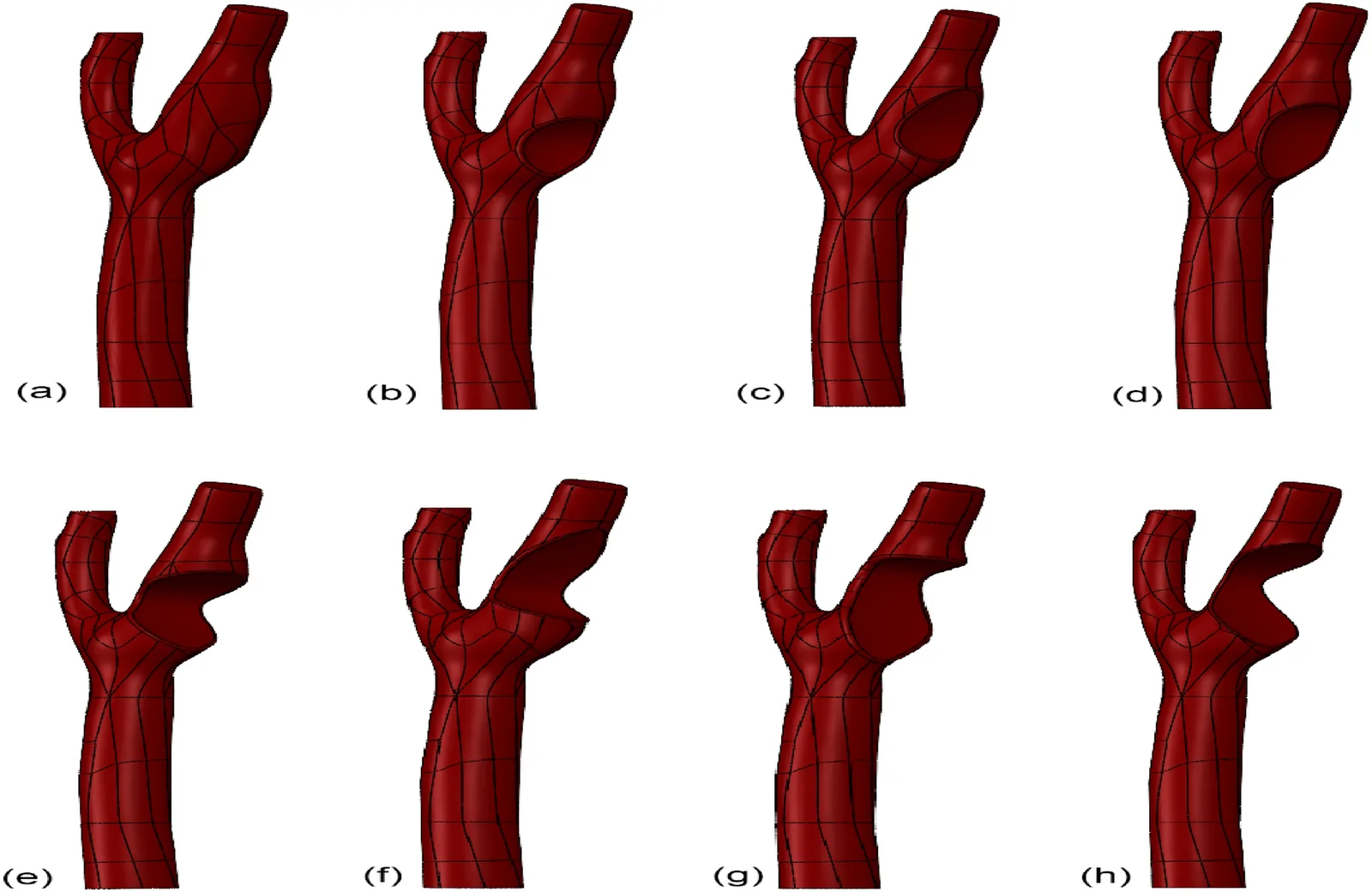
Carotid artery fluid domains, top row—**a** 0%, **b** 30% AOA 0, **c** 30% AOA-1, **d** 30% AOA+1, **e** 60% AOA 0, **f** 60% AOA-1, **g** 60% AOA+1, **h** 80% stenoses

A cosine-shaped stenosis profile is used in this study in order to observe a controlled effect of the geometric changes on flow patterns [10, 31]. The hemodynamics is investigated for 3 levels of sagittal areal occlusion, 30, 60 and 80% and compared with the baseline (0%) case. The orientation of stenosis apex relative to the distal ICA wall is defined as ‘angle of attack (AOA)’. The reference orientation, AOA 0, indicates a nearly normal encroachment of the stenosis with the distal ICA wall, as seen in Fig. 1b. 3 levels of AOA are also investigated, as described in Table 2. It is noted that for the 80% case, SolidWorks was only able to create a watertight geometry for the AOA 0 case. Despite this demerit, it was chosen to retain this case for comparative purposes. The fluid domains for all 8 cases analyzed are shown in Fig. 2. The corresponding solid domains (not shown) were shelled in accordance with the process described in "Stenosis creation" section .

**Table 2:** Description of AOA levels

AOA level	Orientation
AOA 0	Nearly normal to wall
AOA-1	30° upstream with respect to AOA 0
AOA+1	30° downstream with respect to AOA 0

### Meshing and grid convergence study

**Fig. 3:**
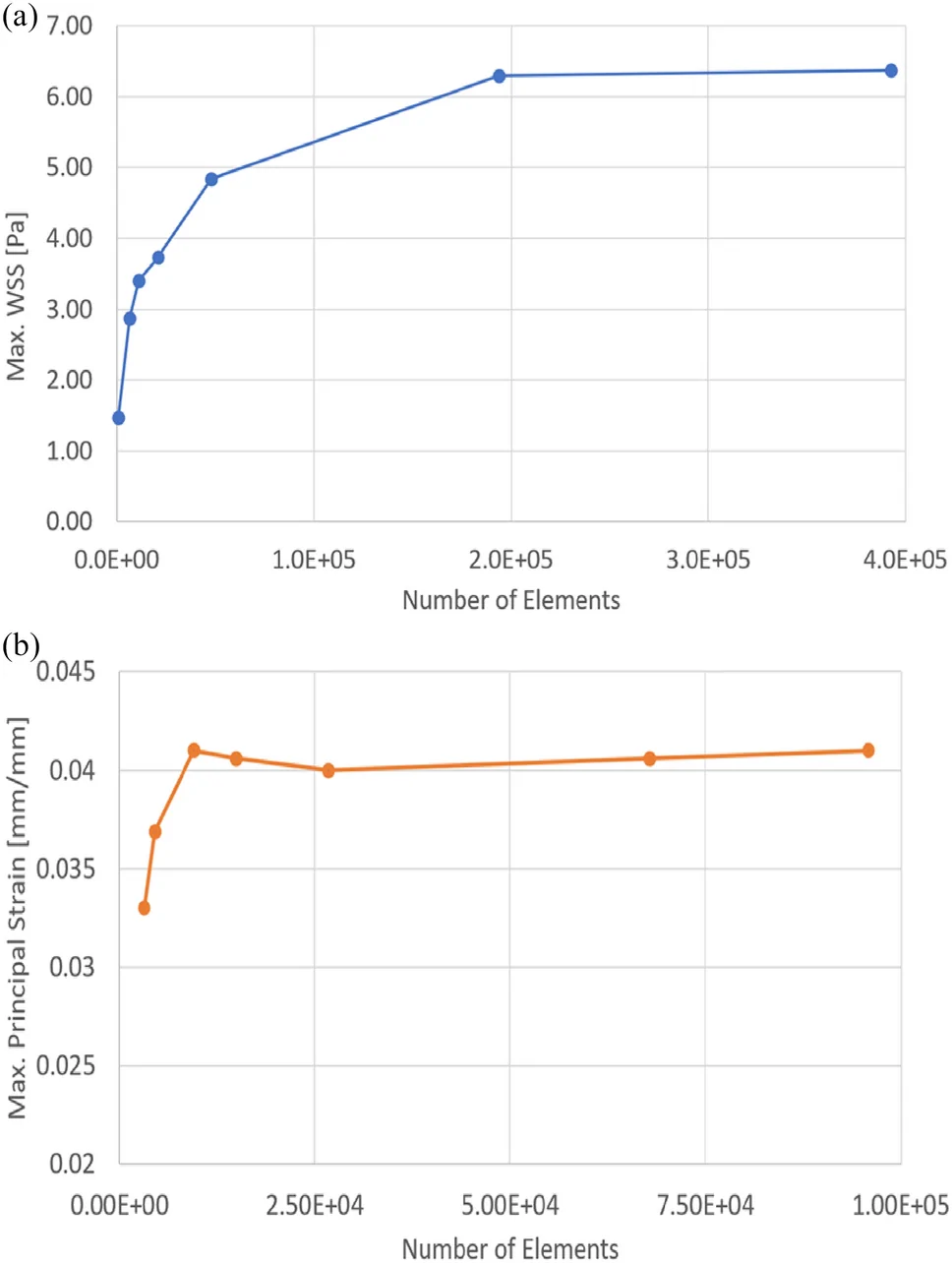
Grid convergence study for the **a** CFD mesh and **b** FEA mesh

The mesh used for the CFD simulation is composed of tetrahedral elements with a 0.5 mm average edge length for each mesh element. 10 wedge-shaped inflation layers were added to the mesh with a target *y+=1*. A grid convergence study was conducted to track changes in maximum WSS versus number of elements, as shown in Fig. 3a. A steady-state solver was used for the grid convergence study, with constant velocity boundary conditions listed in Table 3, while all other solver settings are as mentioned in "Methods for CFD analyses" section .

**Table 3:** Constant velocity boundary conditions used for the grid convergence study

$V_{CCA}$ [m/s]	$V_{ICA}$ [m/s]	$V_{ECA}$ [m/s]
0.165	0.171	0.130

Similarly, for the FEA mesh, a 0.5 mm mesh element size with tetrahedral elements was used. A local sizing of 0.4 mm was applied within a 2 mm radius sphere centered at the carotid sinus in order to improve mesh skewness in the sinus region. A grid convergence study was conducted with a static structural solver to track changes in maximum principal elastic strain versus number of elements, and the results are reported in Fig. 3b. A constant pressure load of 475.13 Pa was applied for the grid convergence study. The material model used is as mentioned in "Methods for FEA analyses" section. For each of the FEA simulations, mesh defeature size was varied as needed to improve mesh skewness.

### Methods for CFD analyses

CFD simulations were performed in ANSYS Fluent 2024 R1 using an incompressible flow model for the Navier–Stokes equations (Eqs. 1 and 2), where *ρ* is the fluid density, $u_i$ is the fluid velocity component, *P* is the pressure, $\tau_{ij}$ is the shear stress component.

1 $$\begin{aligned} & \frac{\partial u_i}{\partial x_i} = 0 \qquad \text {(Continuity)} \end{aligned}$$

2 $$\begin{aligned} & \rho \left( \frac{\partial u_i}{\partial t} + u_j \frac{\partial u_i}{\partial x_j} \right) = -\frac{\partial P}{\partial x_i} + \frac{\partial \tau _{ij}}{\partial x_j} \qquad \text {(Momentum)} \end{aligned}$$

A pressure-based transient solver was employed with the k - *ω * SST turbulence model, with *low-Re* correction and curvature correction. The model is a hybrid Reynolds-Averaged Navier Stokes (RANS) formulation that blends near-wall accuracy with numerical stability under freestream conditions. It introduces a shear stress limiter to improve predictions for adverse pressure gradients and flow separation regions. For more information on its formulation, the reader is referred to [32].

An incompressible, Newtonian rheology model for blood is used with a constant density of $\rho = 1060\ \text [kg /m^{3}]$ and constant viscosity $\mu = 0.0035\ \text [kg/ms]$. As stated in literature, the Newtonian assumption is acceptable for large diameter arteries like the carotid artery [13, 14, 15, 16, 21]. Inlet turbulence internsity was assumed at 5% while outlet turbulence intensity was assumed at 10% for both outlets. The solution numerical methods employed are first-order upwind spatial discretization for momentum, second-order upwind spatial discretization for k and *ω *, second-order for pressure, and first-order implicit transient formulation.

**Fig. 4:**
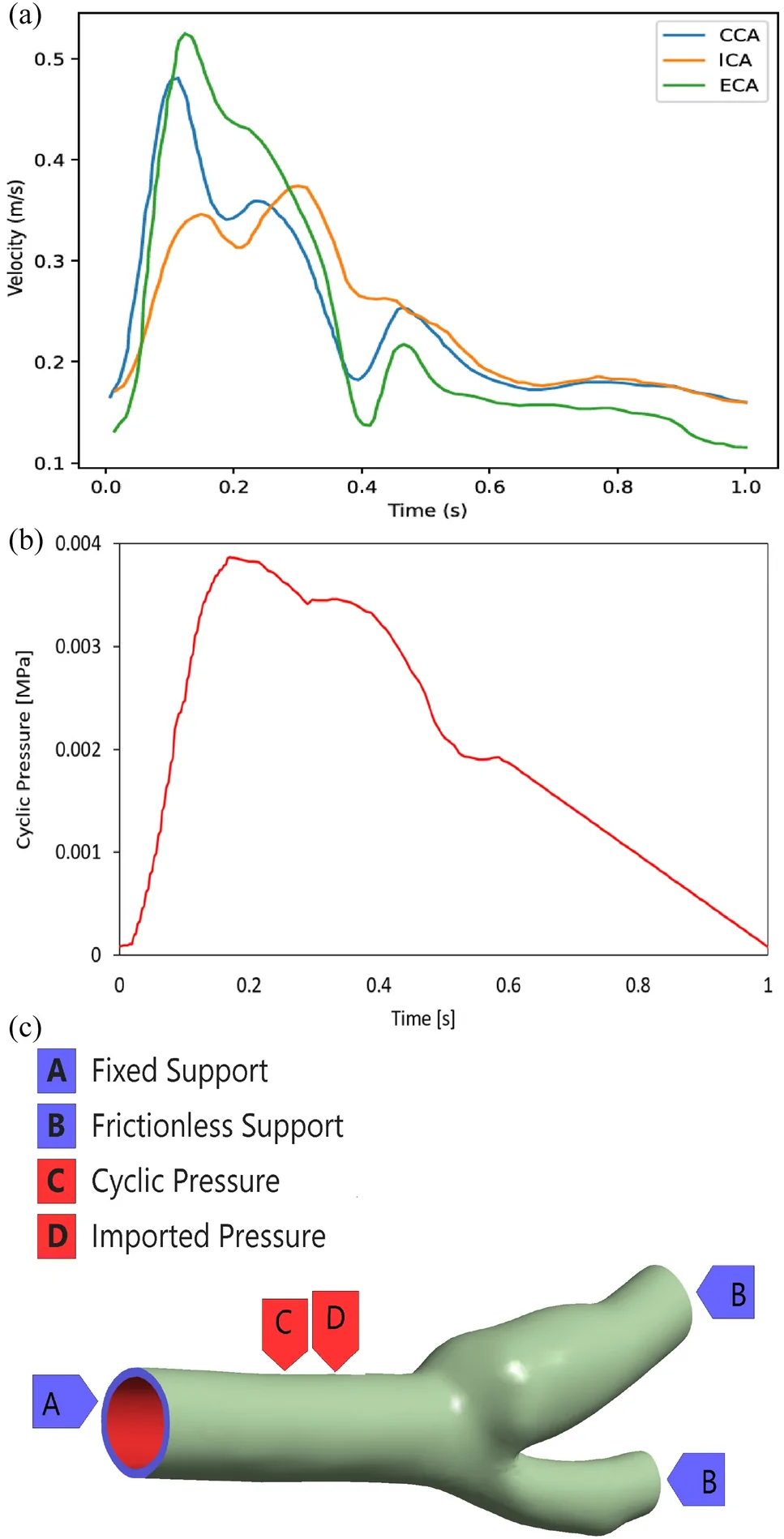
Boundary conditions used in the study **a** inlet and outlet velocities for the CFD simulation (reproduced with permission from [25], © 2024 Elsevier Masson SAS. All rights reserved) **b** cyclic pressure curve for the FEA simulation (adapted from [35]) and **c** schematic of loads and supports for the FEA simulation

Velocity boundary conditions are obtained from pulsed Doppler measurements by Augst et al. (2003) [33], and have been used by [25]. The flow profiles mentioned in Fig. 4a are imposed on the CCA inlet, and ECA-ICA outlets. The threshold convergence for the RMS Scaled residuals of continuity, x, y, z velocities, k, *ω * are set to 1e-6. The time-stepping regime was divided into 2 portions, with a step size of 0.00385 sec from t=0.0 sec to t=0.4 sec (end of systole), followed by a step size of 0.01818 sec from t=0.4 sec to t=1.0 sec (end of diastole). A total of 137 time steps were thus executed over 1 cardiac cycle. The dual time-stepping regime is justified due to the presence of high temporal acceleration and momentum fluxes during the systole, meaning that a finer step size is required to accurately capture the inertial and viscous forces at play. During the diastolic phase, the temporal driving gradients are much gentler, allowing a larger timestep whilst maintaining convergence and stability.

For the simulation of the 80% stenosis case, it is essential to note that the mass flow rate drops considerably at high stenosis levels [34], and imposing the same velocity boundary conditions taken from a healthy subject’s Doppler ultrasound onto a severely stenosed case will lead to non-physiologic values. Therefore, in order to maintain simulation realism, the CCA and ICA velocity profiles are scaled by a factor of 0.5 [34]. It is also assumed that the drop in CCA mass flow rate is equal to the drop in the ICA mass flow rate. The ECA mass flow rate is accordingly adjusted to ensure mass continuity.

### Methods for FEA analyses

The FEA simulations were performed in ANSYS Mechanical 2024 R1 with a transient structural solver. The arterial wall density used is 1300 $[kg/m^3]$ [30]. The base of the artery shell was attached to a fixed support while the outlets ends were attached to frictionless supports, enabling free deformations perpendicular to the axis of the artery. Imported pressure from the CFD simulation were sampled at every 10 time steps of the CFD simulation and applied to the inner walls of the artery shell as one of the boundary conditions for the FEA simulation. This accounts for the dynamic pressure of the blood flowing through the artery over a cycle. In addition, elastic deformation of the artery due to cyclic pressure variation during the cardiac cycle is accounted for in the FEA simulation (Fig. 4b). A minimum of 15 and a maximum of 100 substeps were set for each of the 17 load steps to allow the solver to converge [36]. Figure 4c depicts all the loads and supports used for the FEA simulation.

For the above mentioned cyclic pressure boundary conditions, the data was acquired from the study of Teng et al. Figure 3 [35] and was modified to align the systolic peaks and cycle durations with the velocity boundary conditions, using Piecewise Cubic Hermite Interpolating Polynomials (PCHIP). The modified curve is depicted in Fig. 4b. For additional details on the modification steps, the reader is referred to [27]. Since a physiologic artery geometry is already pre-stressed upto baseline diastolic values, hence, the cyclic stress–strain from this baseline are reported in the FEA simulation.

The artery shell material is modeled as a 3-parameter Mooney–Rivlin hyperelastic solid, whose formulation is elucidated in Eq. 3, where **W** is the strain energy density function, **B** is the left Cauchy-Green deformation tensor, $\lambda_i$ are the principal stretch ratios, and J is the Jacobian. J equals 1 for incompressible models like the Mooney-Rivlin. The data used for the material model is obtained from the study of Teng et al. Figure 1E [35], sampled from 21 symptomatic patients’ carotid plaque samples by Teng et al. [35].

3 $$ \begin{aligned} {\mathbf{W}} = \; & C_{{10}} (\overline{{I_{1} }} - 3) + C_{{01}} (\overline{{I_{2} }} - 3) \\ & + \;{\mathrm{C}}_{{{\mathrm{11}}}} (\overline{{{\mathrm{I}}_{{\mathrm{1}}} }} - {\mathrm{3}})(\overline{{{\mathrm{I}}_{{\mathrm{2}}} }} - {\mathrm{3}}){\text{ + }}\frac{{\mathrm{1}}}{{{\mathrm{D}}_{{\mathrm{1}}} }}({\mathrm{J}} - {\mathrm{1}})^{{\mathrm{2}}} \\ {\mathrm{where}}\quad \overline{{{\mathrm{I}}_{{\mathrm{1}}} }} {\text{ = }} & \;{\mathrm{tr}}({\mathbf{B}}){\text{ = }}\sum\limits_{{{\text{i = 1}}}}^{{\mathrm{3}}} {\lambda _{{\mathrm{i}}}^{{\mathrm{2}}} } \\ \overline{{{\mathrm{I}}_{{\mathrm{2}}} }} {\text{ = }} & \;({\mathrm{tr}}({\mathbf{B}}))^{{\mathrm{2}}} - {\mathrm{tr}}({\mathbf{B}}^{{\mathrm{2}}} ) \\ {\text{ = }} & \;\sum _{{\begin{array}{*{20}c} {{\mathrm{i}},{\text{j = 1}}} \\ {{\mathrm{i}} \ne {\mathrm{j}}} \\ \end{array} }}^{{\mathrm{3}}} \lambda _{{\mathrm{i}}}^{{\mathrm{2}}} \lambda _{{\mathrm{j}}}^{{\mathrm{2}}} \\ \end{aligned} $$

The values of the model constants in the formulation are shown in Table 4, and are calibrated by nonlinear least-squares optimization. It is to be noted that the model fitting was done on uniaxial data reported by Teng et al. hence, the constants in Table 4 are only fitted to the uniaxial test data [27]. This may lead to non-unique solutions for the model constants. We recognize this as a limitation of this study, however, to the best of our knowledge, we did not encounter a dataset wherein all-mode mechanical testing (uniaxial, biaxial and pure shear) was sampled from the same cohort.

**Table 4:** Model constants for the 3-parameter Mooney–Rivlin hyperelastic model

$C_{01}$ [kPa]	$C_{10}$ [kPa]	$C_{11}$ [kPa]
− 108.04	153.32	1691

## Results

**Fig. 5:**
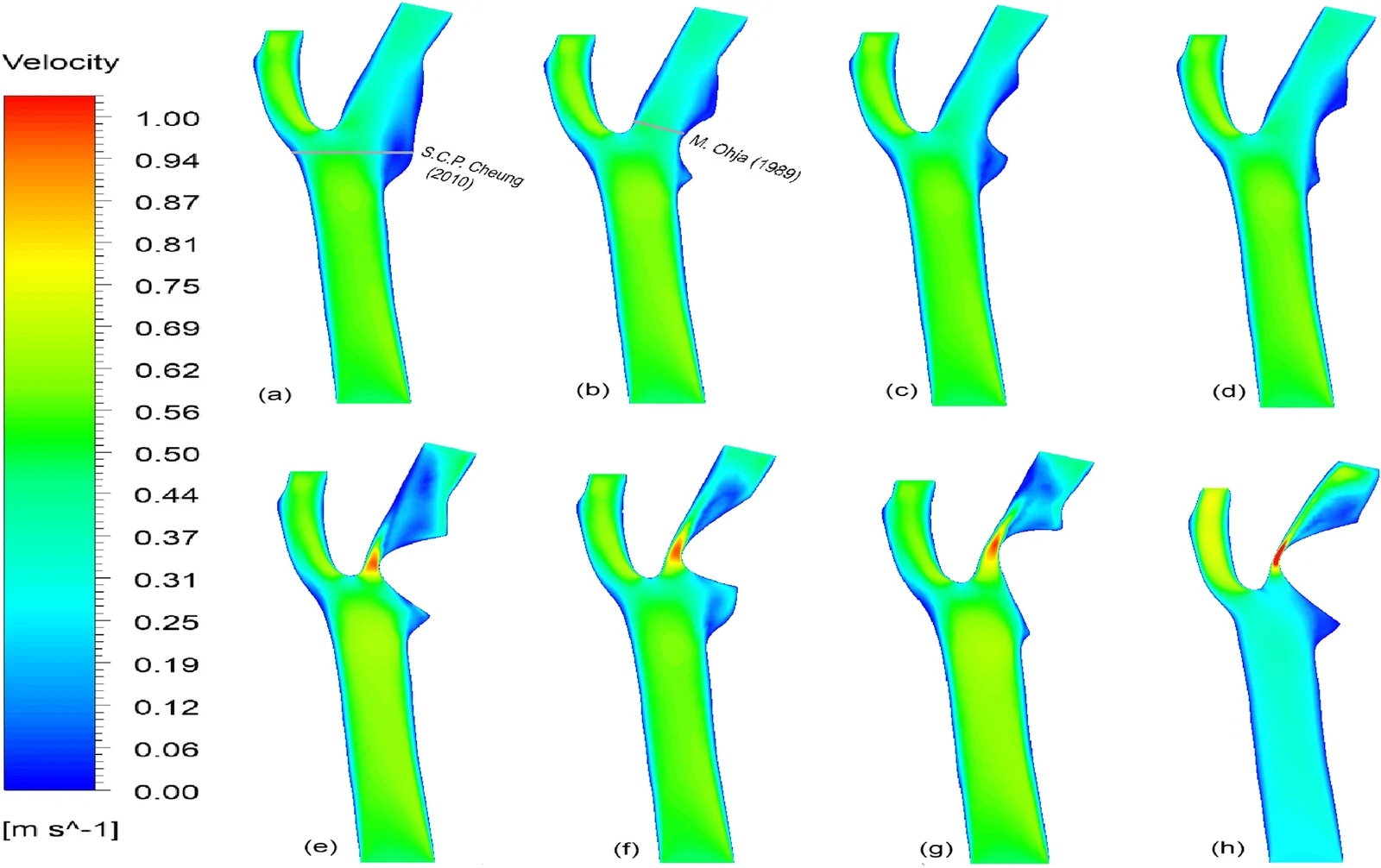
Velocity contours at peak systole (t = 0.173 sec),—**a** 0%, **b** 30% AOA 0, **c** 30% AOA-1, **d** 30% AOA+1, **e** 60% AOA 0,** f** 60% AOA-1, ** g** 60% AOA+1 and** h** 80% stenoses. Sections indicated in grey are selected for comparison with M. Ohja (1989) [12] and S.C.P. Cheung (2010) [37]

The velocity magnitude at the snapshot of peak systole (t = 0.173 sec) are presented in Fig. 5. For validating our model with established literature, we consider the 0% and 30% AOA 0 cases. In the 0% (unstenosed case), the Re at the CCA inlet is 1003 and the centerline velocity in the CCA is 0.50–0.58 m/s, which matches closely with the 0.58 m/s value in Figure 3e of Bit et al. [17], and is also close to the 0.53 m/s max. value in the CCA reported by Shakya et al [25]. Comparing with the work of Yang et al. [38], who use velocity inflow boundary conditions along with pressure outflow boundary conditions derived from a 3-element lumped Windkessel, we find that our ICA centerline velocity is 0.48 m/s at peak systole, whilst their ICA centerline velocity ranges between 0.4–0.6 m/s during systole at comparable diameters (Figure 7a of [38]).

Our work also demonstrates very close agreement with experiments done by Ohja et al. (1989) [12]. In the 30% AOA 0 case, consider the section in grey (Fig. 5b), which has a local Re of 485. The max velocity for this section is 0.48 m/s, which is compared against Figure 5a in [12], which depicts centerline velocity for a 38% asymmetric stenosis. At the same Re, they report a centerline velocity of 0.51 m/s at the center of their stenosis.

**Fig. 6:**
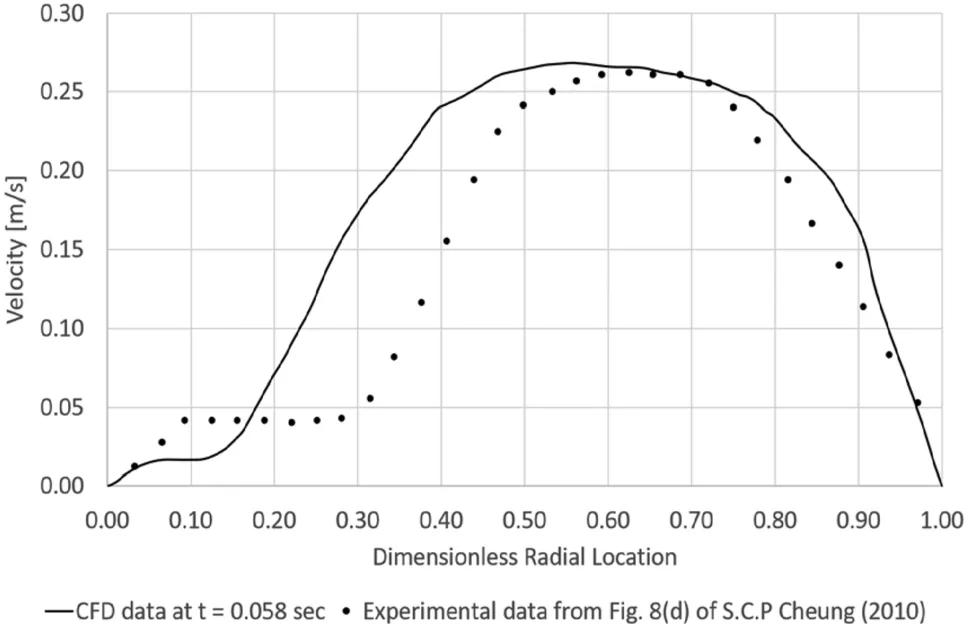
Comparison of velocity profiles from S.C.P. Cheung (2010) [37]. ICA distal wall corresponds to 0 on x-axis

Furthermore, we also demonstrate good agreement with the PIV measurements done by Cheung et al. (2010) [37]. We examine the grey section shown in Fig. 5a, but at a time snapshot of t = 0.058 sec where the inlet Re is 530. It is noted that their PIV study was conducted at a Re of 485. The CFD velocity at this section is compared against Figure 8d in [37], and the profiles are plotted in Fig. 6. It can be seen that our model predicts a wider inertial core and a smaller recirculation zone than their experiments. These discrepancies may be partly attributed to inherent topological differences in their geometry and ours.

**Fig. 7:**
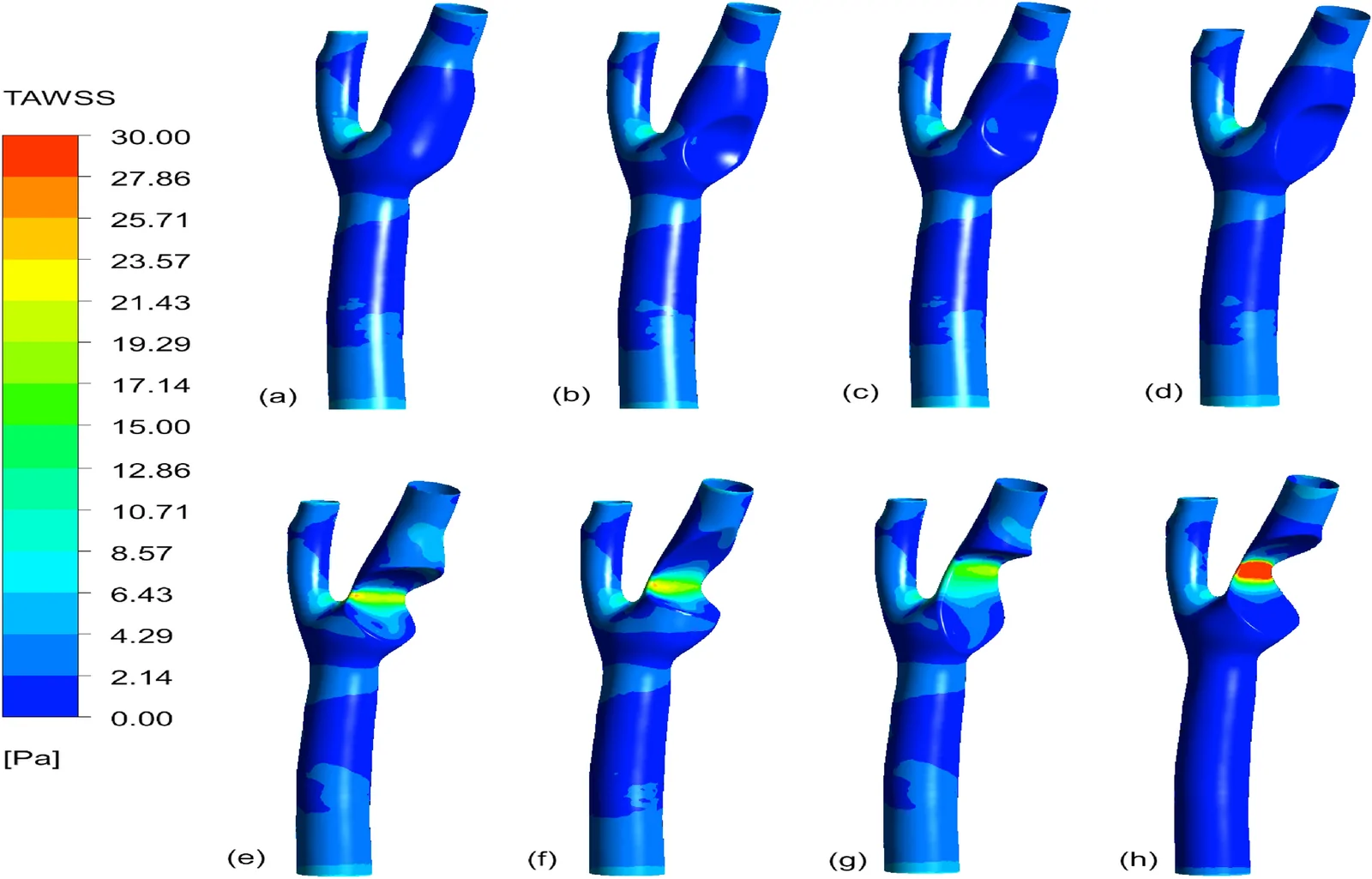
TAWSS contours—**a** 0%, **b** 30% AOA 0, **c** 30% AOA-1, **d** 30% AOA+1, **e** 60% AOA 0, **f** 60% AOA-1, **g** 60% AOA+1, and **h** 80% stenoses

Figure 7 presents magnitude of Time-Averaged Wall Shear Stress (TAWSS) over the cardiac cycle, with its definition in Eq. 4, where $\tau_w$ is the wall shear stress and *T* is the cardiac cycle duration.

4 $$\begin{aligned} \text {TAWSS or }\overline{\tau _w} = \frac{1}{T} \int _0^T |\vec {\tau }_w(t)| \, dt \end{aligned}$$

The TAWSS value near the bifurcation for the unstenosed case is reported between 10–13 Pa, which is compared against the value of 8 Pa reported by Lopes et al. (2019) and Moradicheghamahi et al. (2018) [21, 22], as well as to the value of 10 Pa reported by Kaazempur-Mofrad et al. (2004) and Younis et al (2004) [19, 20]. The differences can be attributed to our use of one-way FSI approach, which is recognized as the prominent limitation of our study.

**Fig. 8:**
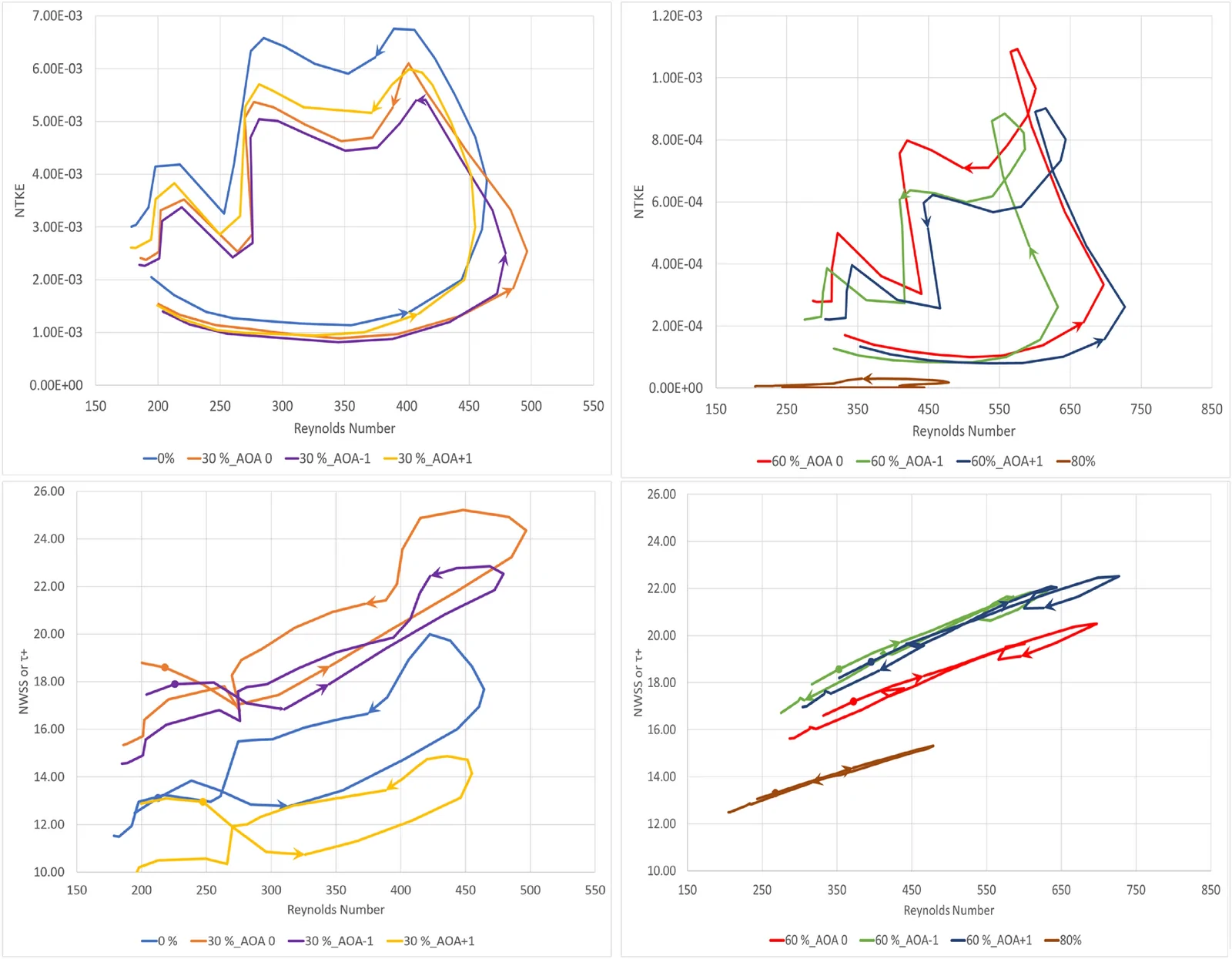
Top Row–NTKE versus Re, and Bottom Row–NWSS versus Re, during the cardiac cycle. Left column—0% and 30% stenoses, Right column—60% and 80% stenoses

In order to characterize risk of disease progression more effectively, it is necessary to shed light on the flow dynamics not just at systole, but also their evolution through time. The quantities of interest must be non-dimensionalized to enable comparison across different nonuniform section geometries. It follows that, we define a nondimensional WSS (NWSS) and nondimensional TKE (NTKE) as stated in Eqs. 5 and 6. The section planes and their peripheries, denoted by $\Gamma _i$ and $\gamma _i$ respectively, and shown in Appendix A. The interplay of these quantities is depicted in Fig. 8.

5 $$\begin{aligned} & \text {NWSS or }\tau _i+ = \frac{\langle |\tau _w|\rangle _{\gamma _i}D_h}{\mu \langle |\textbf{U}| \rangle _{\Gamma _i}} \end{aligned}$$

6 $$\begin{aligned} & \text {NTKE or }k_i+ = \frac{\langle k\rangle _{\Gamma _i}}{\left( \langle |\textbf{U}| \rangle _{\Gamma _i}\right) ^2} \end{aligned}$$

where* D*_h_ is the hydaulic diameter at plane $\Gamma _{i},$

$\langle |{\mathbf{U}}|\rangle _{{\Gamma _{i} }}$ is the bulk velocity across $\Gamma _{i},$

$\langle k\rangle _{{\Gamma _{i} }}$ is the mean TKE across $\Gamma _{i},$

$\langle |\tau _{w} |\rangle _{{\gamma _{i} }}$ is the mean WSS along curve $\gamma _i$

**Fig. 9:**
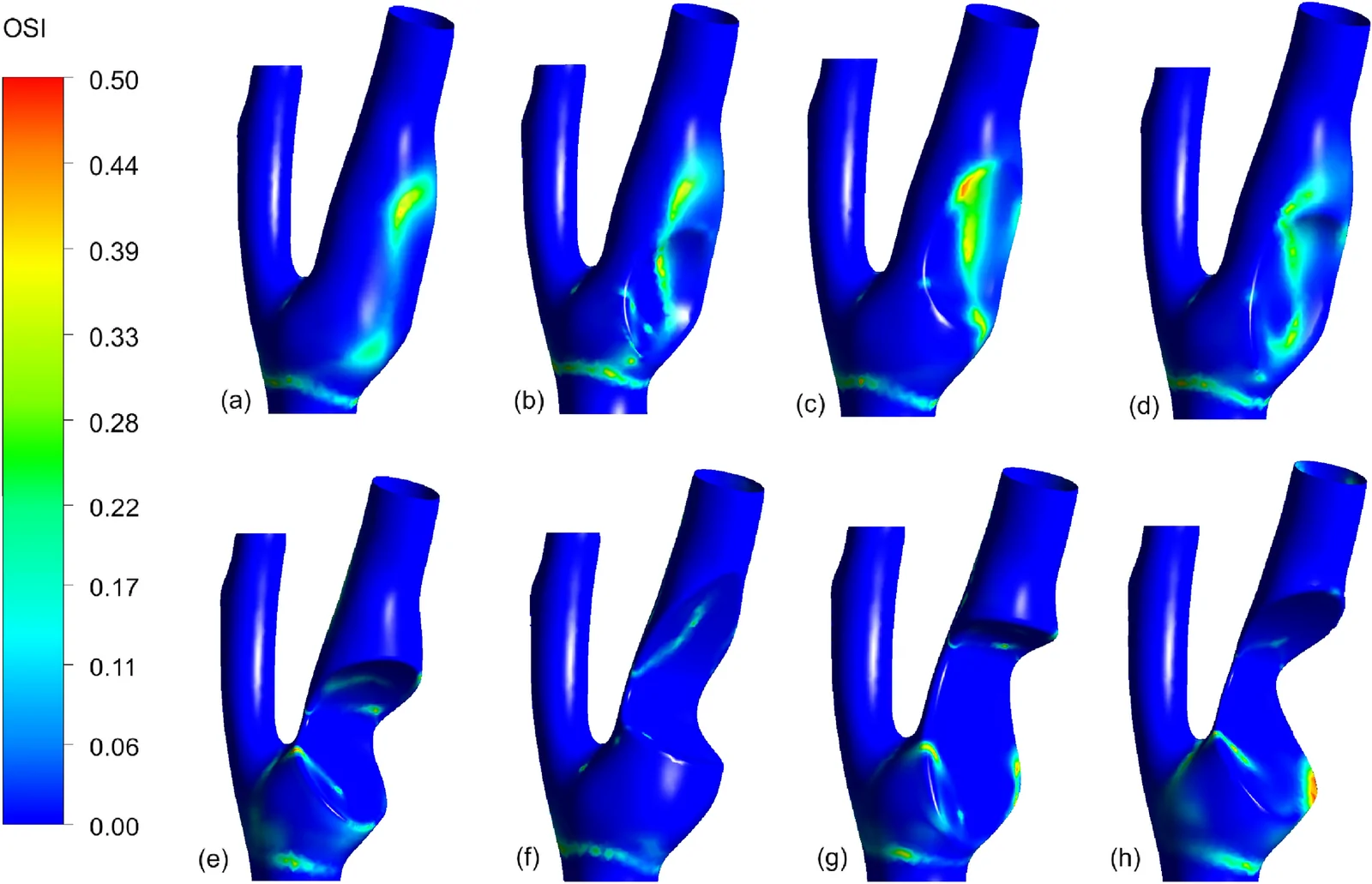
OSI contours—**a** 0%, **b** 30% AOA 0, **c** 30% AOA-1, **d** 30% AOA+1, **e** 60% AOA 0, **f** 60% AOA-1, **g** 60% AOA+1 and **h** 80% stenoses

Figure 9 represents the Oscillatory Shear Index (OSI) over the cardiac cycle, with its definition in Eq. 7.

7 $$\begin{aligned} \text {OSI} = 0.5\left( 1 - \frac{|\int _0^T \vec {\tau }_w(t) \, dt|}{\int _0^T |\vec {\tau }_w(t)| \, dt}\right) \end{aligned}$$

**Fig. 10:**
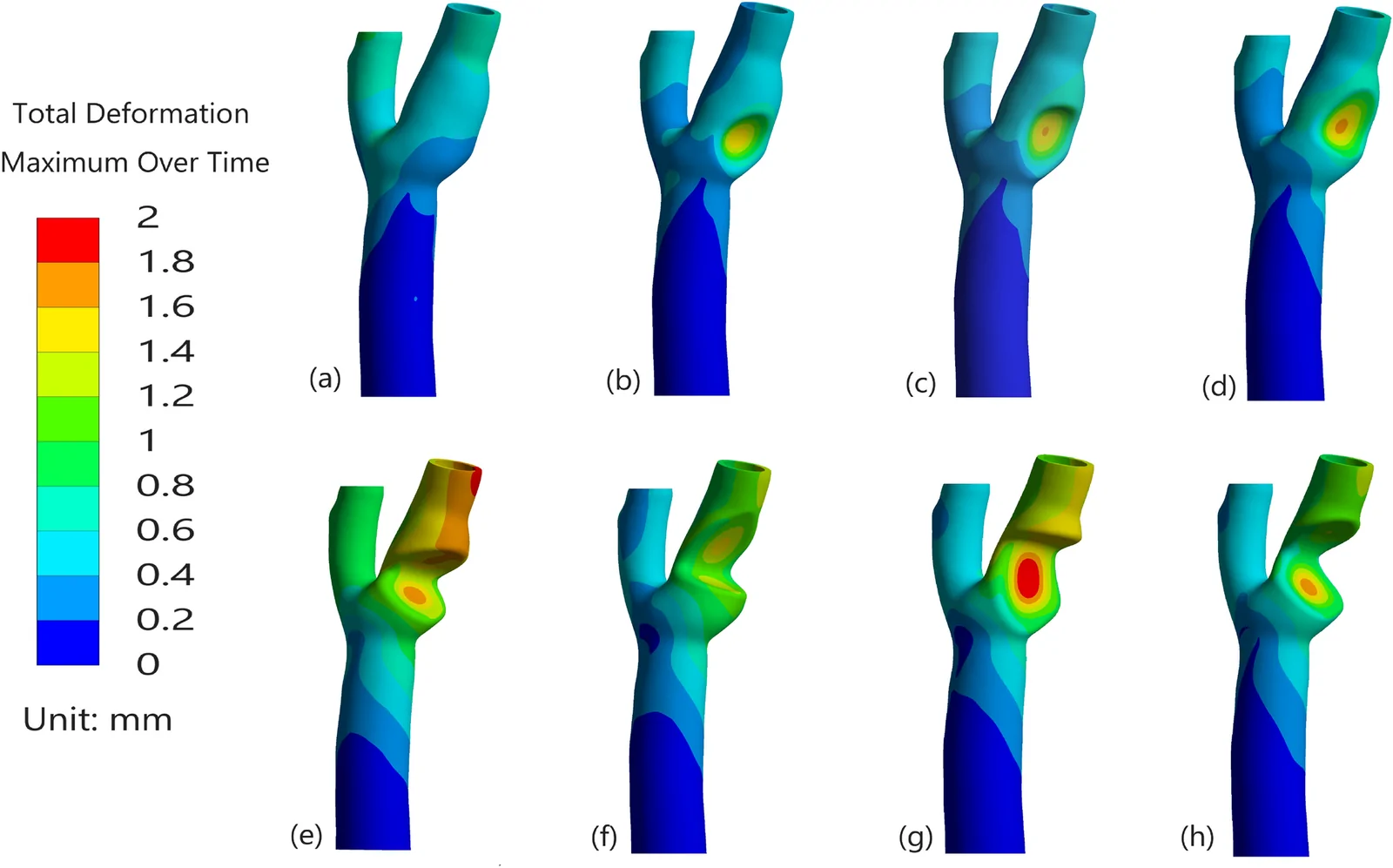
Total deformation of the arterial wall,—**a** 0%, **b** 30% AOA 0, (**c** 30% AOA-1, **d** 30% AOA+1, **f** 60% AOA 0, **g** 60% AOA-1 **g** 60% AOA+1 and **h** 80% stenoses

**Fig. 11:**
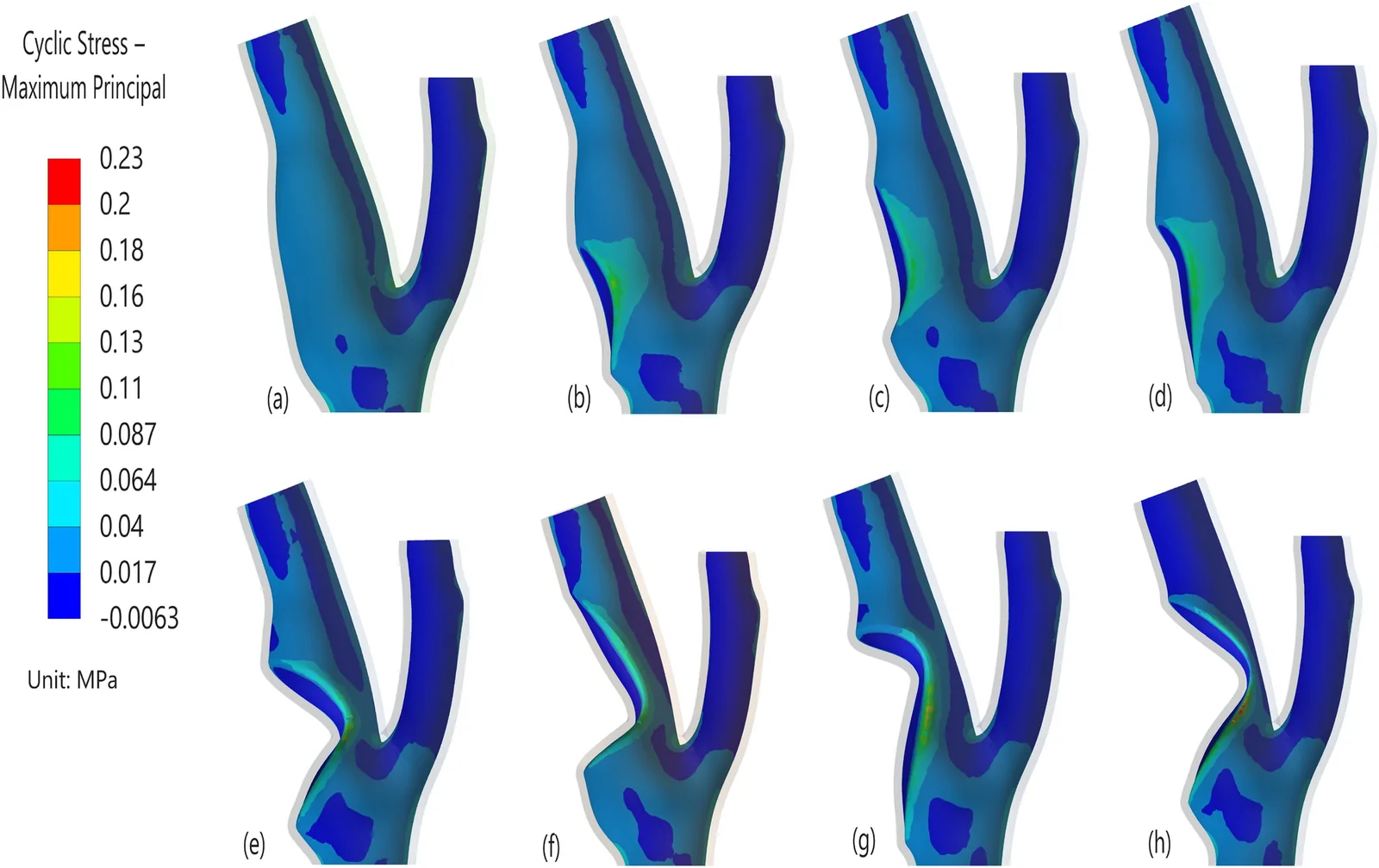
Maximum principal cyclic stress at peak systole,—**a** 0%, **b** 30% AOA 0, **c** 30% AOA-1, **d** 30% AOA+1, **f** 60% AOA 0, **g** 60% AOA-1, **g** 60% AOA+1 and **h** 80% stenoses

Figure 10 depicts the total deformation of the arterial wall, presenting the snapshot where maximum deformation was attained for that case. It is acknowledged that since the study employs 1-way FSI, the arterial wall deformation is likely overpredicted as compared to a physiologic arterial mechanical response. Figure 11 tracks the maximum principal cyclic stress at peak systole. The FEA simulation is validated by comparing the maximum Von-Mises cyclic strain (not shown) on the artery walls at the systolic peak. Across all 8 cases, this value is reported to lie between 0.10–0.14, which matches the work of [20, 39, 40].

## Discussion

The Discussion section is organized into 6 parts. "Velocity contours" section entails discussion on the velocity contours, highlighting the nonlinear flow dynamics involved. "Time-averaged wall shear stress" section talks about Time-Averaged Wall Shear Stress (TAWSS) and identifies low TAWSS regions, prone to be susceptible to atheroprogression. "Interplay between Re, WSS and TKE" section elucidates how the interplay between Re, nondimensional WSS (NWSS) and nondimensional TKE (NTKE) reveals insights on disturbed flow dynamics, while Oscillatory shear index (OSI) section discusses the Oscillatory Shear Index (OSI). "Arterial wall deformation and principal stresses" section includes the structural dynamics observed for the 8 cases and its implications on disease progression. "Qualitative assessment of disease progression" section ultimately coheres the discussion in the previous sections to build a qualitative framework of relative risk of atheroprogression. "Limitations" section addresses the shortcomings of this study.

### Velocity contours

Figure 5 reveals the velocity contours for each case at peak systole (t = 0.173 sec). The velocity profiles in the CCA across cases indicate a broad region of uniform speed flow (plug flow) as the flow profile is still developing. There is a hotspot of slow, recirculating flow at the distal ICA wall for the unstenosed case (Fig. 5b). The flow impinging at the sinus from the CCA can be seen reorienting predominantly toward the proximal (inner) wall in the ICA and ECA, leaving the distal outer wall with comparatively low momentum exchange. The broadening of the vessel’s local diameter at the base of the distal ICA is also seen to influence flow separation. The distal ICA wall will hence have much higher particle residence time, potentially facilitating LDL migration across the intimal layer, which is responsible for atherogenesis. There is also the presence of a shear layer (light blue-green interface) seen in the 0% and 30% cases. This may indicate the presence of turbulence, primarily because of the widening of conduit area slightly prior to the bifurcation. It is observed that even for healthy arteries, geometric features have a significant influence on flow dynamics and at least intermittent turbulence may be present.

Comparing Fig. 5b–d shows that for the 30% stenosis, flow separation has occurred for the AOA-1 and AOA+1 cases upstream of the stenosis, while for the AOA 0 case, there is a brief reattachment at the apex. The largest recirculation pockets around the stenosis are seen for the 30% AOA+1 case. Figure 5e–g show that for the 60% stenosis, all 3 AOA cases behave like a converging throat upstream of the apex, with flow separation immediately downstream of it. For all the 3 cases for 60%, we see that the increased jet velocity encounters a rapidly widening area post the apex, leading to rapid dissipation of the inertial core and a transformation to a large recirculating pocket in the downstream. It is seen that the 60% AOA 0 case indicates the largest recirculation pocket. For the 80% case (Fig. 5h), most of the flow can be seen reorienting into the ECA, leaving a quiescent, recirculating pocket of flow in the post-stenosis ICA region on the distal side and a stenotic jet on the proximal side. Despite a 50% drop in inlet velocity profile for this case (as stated in "Methods for CFD analyses" section ), we see that the peak velocity is 1.49 m/s, almost 2.5 times that of the unstenosed case’s CCA velocity.

### Time-averaged wall shear stress

Figure 7 represents the Time-Averaged Wall Shear Stress (TAWSS) averaged over the cardiac cycle, as defined in Eq. 4. The first millimeter of the inlet and outlets has been hidden from view due to numerical artifacts. Figure 7a indicates that a significantly large region of the unstenosed distal ICA experiences sustained low values of shear stresses (TAWSS *<= 0.4* Pa). A circumferential region of the CCA upstream of the bifurcation, also experiences similarly low TAWSS. These regions have been correlated in the literature as being susceptible to atherogenesis [1, 3, 4, 5, 19, 20]. As reported in several studies, low hemodynamic shear stresses are associated with disorganized endothelial cell shape, inhibition of certain vasoregulatory enzymes, higher oxidative stress, higher inflammatory mediators and lower anti-inflammatory mediators. It is also associated with decreased thrombo-resistance and increased lipid uptake by the endothelial wall [1, 3, 19].

Figure 7b–d reveal that much of the distal ICA wall of the 30% stenosed geometry cases experiences sustained low values of TAWSS similar to the unstenosed case. This suggests that atheroprogression through further plaque thickening is highly likely for the 30% cases. Figure 7e–g show that the 60% stenosed geometries experience wide bands of higher TAWSS across the stenoses’ apex. The 60% AOA 0 case has a max. TAWSS of 26 Pa, the 60% AOA-1 case has a max. TAWSS of 24 Pa, while the 60% AOA+1 case has a max. TAWSS of 20 Pa. The observed maxima can be explained due to the orientation of the acting shear area. When flow impinges on the stenosis, in the AOA-1 case and AOA 0 cases, the shear stress acting area would be oriented cross-stream into the direction of the proximal ICA wall, which will stretch the endothelium further towards the proximal side of the ICA. In contrast, the AOA+1 case stretches the acting shear area cross-stream away from the bulk flow and into the distal ICA wall. It is seen that the 80% stenosis case (Fig. 7h) receives a max. TAWSS of 60 Pa even at 50% damping of CCA and ICA flow rates. Similar high values of TAWSS have been reported in Figure 9 of [41]. Such consistent high shear regions are likely to promote faster endothelial erosion. These findings concur with the work of [6, 26, 41, 42, 43], indicating that high TAWSS is observed at the stenosis throat in advanced stages. It is also emphasized that the the risk of adverse cardiovascular events is higher at severe stenoses [7, 44]. Yang et al. [26] also argue that a positive correlation exists between high TAWSS and advanced-stage progression. However, the exact physiological mechanism behind this remains poorly understood.

### Interplay between Re, WSS and TKE

The top row in Fig. 8 presents the relationship between NTKE and NWSS versus Re for the section planes $\Gamma _i$. The flow of physical time is marked by the direction of the arrows shown. It is striking from Fig. 8 (top left) that the relationship for 0% and 30% cases is hysteretic. NTKE is low and nearly constant in early systole, but steeply rises as Re starts decreasing from peak systole onward. This rapid rise is followed to an oscillating pattern of TKE throughout the diastole. Disturbed flow dynamics have been known to favor atheroprogression [45, 46]. The presence of shear layers and recirculation zones discussed in "Velocity contours" section means that TKE can influence energy exchange in the direction of the wall. Furthermore, given that these fluctuations in NTKE are basically temporal fluctuations in space-averaged turbulence (Eq. 6), they modulate the near-wall momentum transport and therefore contribute to the observed cyclic fluctuations in the space-averaged wall shear stress.

In milder stenosis cases, the conduit diameter is large enough for a complex interplay between NTKE and oscillatory viscous effects. This interplay is being seen in Fig. 8 (bottom left), with all 4 cases exhibiting a viscous, oscillatory dominance during post systole, marked by a higher NWSS on Re-descent than during the Re-ascent stage. The area enclosed by this counter-clockwise loop reflects the relative duration and magnitude of this delayed response, and may be indicative of the persistence of flow disturbances or unsteadiness-induced instabilities.

For the more severe cases of stenosis, we see that though there a hysteretic relationship between NTKE and Re, the small conduit diameter means that inertial forces overpower in this region. Viscous effects do not play a major role, and NWSS loading stays nearly linear with Re. Hence, the flow disturbed flow characteristics were not persistent throughout the cycle for the high stenoses cases. However, the risk of MACE events for more severe stenosis still remains high. Furthermore, high TAWSS combined with flatter NWSS curves may still influence endothelial injury or plaque destabilization [7, 44].

### Oscillatory shear index (OSI)

To lend further support to the above discussion, we present contours of Oscillatory Shear Index (OSI) in Fig. 9. It can be seen that areas of high OSI are markedly present in the ICA distal wall for the unstenosed case, and throughout the surface area of the stenoses for the 30% cases. the 30% AOA-1 case is seen to exhibit the highest OSI with a wide band across the stenosis. It is closely followed by the AOA 0 and AOA+1 cases. The observed behavior correlates with the expected flow behavior around obstructions. The AOA-1 case, with its apex oriented into the flow, promotes flow detachment and possible vortex formation at its apex. In contrast, the AOA 0 and AOA+1 cases may delay it because of their geometric orientation.

For the 60% cases, we observe that OSI signatures is minimal. This means that the local shear patterns exhibit minimal deviation from the global flow regime prescribed by the boundary conditions. Furthermore, it is worth noting that the unsteadiness elucidated in the NWSS versus Re curves correlate well with OSI across all 8 cases. This indicates that they may be helpful in assessing athero-promoting flow signatures as well. While OSI remains the metric of choice in identifying regions of oscillatory shear, it may not necessarily capture the underlying coupling between turbulence diffusion and wall shear. The NTKE and NWSS curves at a section are therefore presented as a complementary viewpoint that reveals how geometry-dependent turbulence fluctuations modulate the shear fluctuations in time. They may be particularly helpful in cases where similar OSI values can arise from different unsteady mechanisms.

### Arterial wall deformation and principal stresses

Figure 9 reveals insights about the maximum deformation of the artery walls from the FEA simulation. It is noted that the maximum deformations of the geometries occur at different times. The deformation waveform (not shown) exhibits some transient oscillations, due to a combination of supports, inertia, damping effect of the structure, as well as nonlinear stiffness due to the hyperelasticity. They may also be indicative of excitations near the natural frequency the system, which has been reported in prior literature [47].

Furthermore, by confining the structural simulation to the luminal boundary, we isolate the geometric effects of the stenosis on the wall mechanics. A prominent focus is observed in the posterior of the CCA (not shown) that is a hotspot of high deformation. The max. deformation is 0.86 mm for the unstenosed case. It is caused mainly by the flow reoriented backwards from the bifurcation mixing with the flow coming from the inlet. For the 30% cases (Fig. 9b–d), the maximum deformation is seen at the center of the stenosis. For the 60% cases (Fig. 9e–g), the region of high deformation on the stenosis is larger. Hotspots of high deformation may increase risk of an intraplaque hemorrhage with repeated cardiac cycles [48, 49].

For the 60% AOA 0 case, a peak deformation of 1.8 mm is observed on one side of the stenosis. Whereas, the AOA-1 case displayed two foci of deformation around 1.6 mm on both sides of the stenosis. The 60% AOA+1 case represents the largest hotspot of deformation at 2.1 mm. This is best explained by the combination of flow impingement, inertia and damping effect exhibited by that orientation of stenosis This may increase the risk of intraplaque hemorrhage for the 60% cases. The 80% case is also similarly vulnerable to intraplaque hemorrhage.

The maximum strain is occurring at the systolic peak due to accurate interpolated pressure alignment. Figure 10 reveals the maximum principal cyclic stress at peak systole for the 8 geometries. It is seen that 5 out of 8 (Fig. 10a–d,f) geometries exhibit a maximum principal stress of 0.14 MPa. The carotid sinus and the stenosis edges where it meets the artery experience the most principal stress. This may be due to the shoulders of the plaque acting as geometric inflection points. The 60% AOA 0 case and the AOA+1 case (Fig. 10e,g) experience a maximum principal stress of 0.18 MPa, which closely matches the plot of Teng et al. Figure 6D for a 3D fully coupled FSI simulation [35]. It is to be noted that the 80% case experiences a maximum principal stress of 0.23 MPa. This is close to the established 0.3 MPa rupture threshold. However, as noted by [24], additional work needs to be done to unravel the failure modes and refine the threshold with location and tissue-specific insights.

### Qualitative assessment of disease progression

**Table 5:** Relative risk of plaque progression based on hemodynamics

Rank	Geometry	Reasoning
1	30% AOA-1	Moderate region of low TAWSS, highest OSI with a wide band across the stenosis surface
2	0%	Largest region of low TAWSS, moderate band of OSI, large area under NWSS versus Re curve, highly athero-promoting from a hemodynamics perspective
3	30% AOA 0	Moderate region of low TAWSS, moderate OSI band, moderate area under NWSS versus Re curve
4	30% AOA+1	Large region of low TAWSS, moderate OSI band, marginally smaller area under NWSS versus Re
5	60% AOA-1	High TAWSS at stenosis (24 Pa), two prominent foci of high deformation on stenosis, risk of endothelial erosion or possible intraplaque hemorrhage
6	60% AOA 0	High TAWSS at stenosis (26 Pa), two prominent foci of high deformation colocalizing at stenosis and sinus, risk of endothelial injury or possible intraplaque hemorrhage
7	60% AOA+1	High TAWSS at stenosis (20 Pa), largest magnitude focus of high deformation on stenosis, risk of endothelial injury or possible intraplaque hemorrhage
8	80%	Very high TAWSS at stenosis (60 Pa), high risk of endothelial erosion or possible plaque rupture rather than further progression

**Table 6:** Relative risk of plaque vulnerability for advanced stenoses

Rank	Geometry	Reasoning
1	80%	Very high TAWSS at stenosis (60 Pa), focus of high deformation (1.8 mm) on stenosis, highest principal stress, high risk of endothelial erosion or possible plaque rupture
2	60% AOA+1	High TAWSS at stenosis (20 Pa), largest magnitude of high deformation (2.1 mm) on stenosis, risk of endothelial erosion or possible intraplaque hemorrhage
=3	60% AOA-1	High TAWSS at stenosis (24 Pa), two prominent foci of high deformation on stenosis, risk of endothelial erosion or possible intraplaque hemorrhage
=3	60% AOA 0	High TAWSS at stenosis (26 Pa), two prominent foci of high deformation on both sides of stenosis, risk of endothelial erosion or possible intraplaque hemorrhage

Based on the analysis of the fluid-structural mechanics discussed above, it is of clinical interest to provide a qualitative assessment of the relative risk of plaque progression in each of the 8 artery geometries, and is elucidated in Table 5. Table 6 describes the mechanical vulnerability of the stenosis for the advanced stenosis stages. It is essential to note that atherosclerosis is a multi-factorial disease involving lifestyle causes as well as other patho-physiological risk factors, hence a causative association cannot be established.

### Limitations

The most prominent limitation of the study was that we incorporated 1-way FSI, not 2-way FSI. 2-way FSI was attempted, however, due to the fluid and structural densities being similar, and the material model being highly compliant and nonlinear, numerical instabilities with the current framework prevented a converged solution. Another limitation which was also declared in "Methods for FEA analyses" section is that the parameter set of the Mooney–Rivlin solid may be non-unique due to lack of an all mode deformation dataset. Lastly, the strength of the qualitative assessment of the relative risk of progression is limited by the large increments of stenotic severity employed.

## Conclusion

This study investigated the combined influence of stenosis severity and orientation on the hemodynamics and wall that affect atheroprogression. It is concluded that our 1-way FSI model sits at the upper-bound of physiologic athero-promotive reality, by virtue of the validation of the velocity, TAWSS, cyclic strain and max. principal cyclic tress metrics against published experimental and computational works. Low TAWSS zones which are known to be favorable to atheroprogression, were identified. Hysteretic signatures of NWSS with Re yield insights in the persistence of flow disturbances. It was observed that the 0% and 30% geometries exhibited the highest amounts of oscillatory shear while 60% and 80% geometries recorded minimal oscillatory shear. The prevalence of the hysteretic signatures of NWSS correlated well with the regions of high OSI. The structural simulations revealed regions at risk of intraplaque hemorrhage or rupture. The effect of angular orientation yielded mixed results. For the milder stenoses cases, the AOA-1 case exhibited higher amounts of disturbed flow signatures and hence, may be at more risk of progression relative to the AOA 0 and AOA+1 case. In contrast, for the advanced stenosis cases, the AOA+1 seemed most at risk of destabilization compared to the AOA 0 and AOA-1 cases. Finally, the 8 geometries were ranked for atheroprogression risk based on the hemodynamics and wall mechanics investigated. In summary, the study highlights the influence of geometric features like stenosis severity and orientation in shaping the relative risk of atheroprogression.

## Figures and Tables

**Fig. 12:**
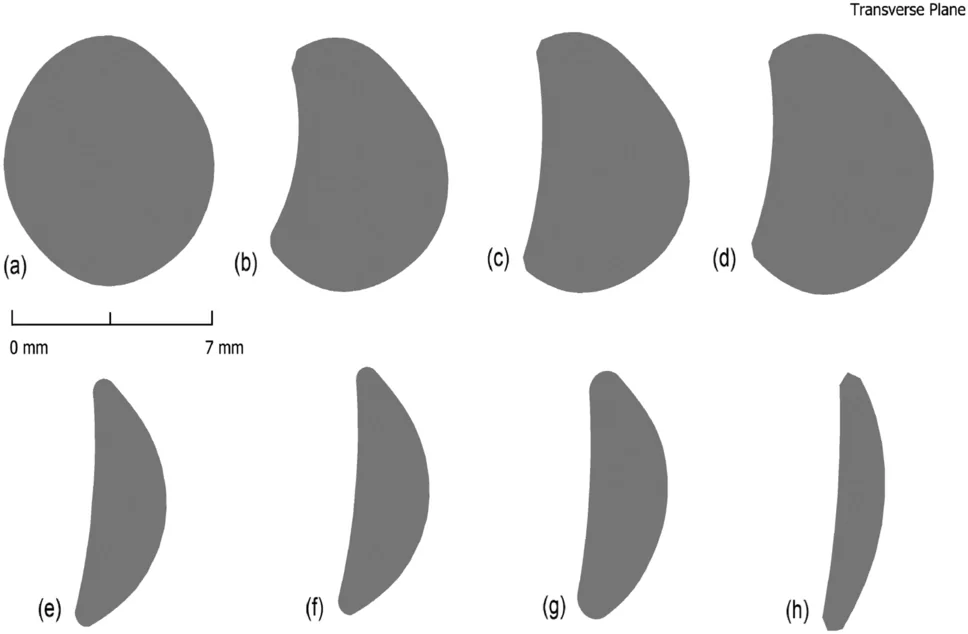
Section planes $\Gamma _i$ selected for the NTKE, NWSS interplay—**a** 0%, **b** 30% AOA 0, **c** 30% AOA-1, **d** 30% AOA+1 **e** 60% AOA 0, **f** 60% AOA-1, **g** 60% AOA+1 and **h** 80% stenoses

## Data Availability

Data will be made available upon reasonable request.

## Section planes $\Gamma _i$

The section planes $\Gamma _i$ used in the NWSS and NTKE analysis are depicted in Fig. 12. Bounded curves $\gamma _i$ represent the periphery of these planes.
